# Characterization of Lung Injury following Abrin Pulmonary Intoxication in Mice: Comparison to Ricin Poisoning

**DOI:** 10.3390/toxins14090614

**Published:** 2022-09-02

**Authors:** Anita Sapoznikov, Yoav Gal, Ron Alcalay, Yentl Evgy, Tamar Sabo, Chanoch Kronman, Reut Falach

**Affiliations:** Department of Biochemistry and Molecular Genetics, Israel Institute for Biological Research, Ness-Ziona 74100, Israel

**Keywords:** abrin, ricin, intranasal, lungs, alveolar epithelial type II cells, neutrophils, alveolar–capillary barrier, junction proteins, glycocalyx

## Abstract

Abrin is a highly toxic protein obtained from the seeds of the rosary pea plant *Abrus precatorius*, and it is closely related to ricin in terms of its structure and chemical properties. Both toxins inhibit ribosomal function, halt protein synthesis and lead to cellular death. The major clinical manifestations following pulmonary exposure to these toxins consist of severe lung inflammation and consequent respiratory insufficiency. Despite the high similarity between abrin and ricin in terms of disease progression, the ability to protect mice against these toxins by postexposure antibody-mediated treatment differs significantly, with a markedly higher level of protection achieved against abrin intoxication. In this study, we conducted an in-depth comparison between the kinetics of in vivo abrin and ricin intoxication in a murine model. The data demonstrated differential binding of abrin and ricin to the parenchymal cells of the lungs. Accordingly, toxin-mediated injury to the nonhematopoietic compartment was shown to be markedly lower in the case of abrin intoxication. Thus, profiling of alveolar epithelial cells demonstrated that although toxin-induced damage was restricted to alveolar epithelial type II cells following abrin intoxication, as previously reported for ricin, it was less pronounced. Furthermore, unlike following ricin intoxication, no direct damage was detected in the lung endothelial cell population following abrin exposure. Reduced impairment of intercellular junction molecules following abrin intoxication was detected as well. In contrast, similar damage to the endothelial surface glycocalyx layer was observed for the two toxins. We assume that the reduced damage to the lung stroma, which maintains a higher level of tissue integrity following pulmonary exposure to abrin compared to ricin, contributes to the high efficiency of the anti-abrin antibody treatment at late time points after exposure.

## 1. Introduction

The family of ribosome-inactivating proteins (RIPs) groups all enzymes (EC.3.2.2.22) with a so-called RIP domain which comprises N-glycosylase activity and enables these proteins to catalytically inactivate ribosomes. The highest number of plant RIPs has been found in angiosperm plants [1,2,3,4], including fungi [3,5,6], algae [7] and bacteria [8]. Structurally, plant RIPs can be divided into two main groups, depending on the presence or absence of a quaternary structure. Type 1 RIPs (~30 kDa) are single-chain proteins with enzymatic action, whereas type 2 RIPs (~60 kDa) consist of an enzymatically active A-chain linked to a B-chain with lectinic properties through a disulfide bridge. The B-chain binds to carbohydrates on the cell surface, allowing A-chain cell internalization. The absence of a lectinic chain prevents type 1 RIPs from binding to the cell, which are consequently less toxic with respect to type 2 RIPs, due to the difficulty of entering the cell [3]. Moreover, a third group of RIPs, known as type 3 RIPs, consists of a type-1-like N-terminal domain with N-glycosylase activity, covalently linked to a C-terminal domain with an unknown function [9].

The toxins abrin and ricin produced from the seeds of *Abrus precatorius* and *Ricinus communis*, respectively, are classified as type II RIPs and consist of an enzymatically active A-chain disulfide linked to a B-chain. The B-chain is a galactose-specific lectin that is responsible for the binding of toxins to glycoproteins or glycolipids on the surface of cells to promote endocytosis of the toxin [10]. Receptor-dependent internalization of the toxins involves retrograde transport to the endoplasmic reticulum, where the disulfide bond connecting the A and B subunits is reduced [11], allowing the release of the catalytically active A-chain into the cytoplasm [12]. The A subunit of both toxins is an RNA N-glycosylase that catalyzes the site-specific release of an essential adenine moiety, located in a highly conserved stem–loop within the small subunit of the ribosome 28S rRNA [13,14,15]. The irreversible depurination of the stem–loop by the A subunit prevents the binding of elongation factors to ribosomes, thereby inhibiting protein synthesis and eventually causing cell death [16,17].

Abrin’s potential use as a chemical weapon stems from its high toxicity, together with the fact that it can be isolated from jequirity beans at a low cost by a relatively simple procedure. The relatively low-scale cultivation of jequirity plants compared with *Ricinus* (castor oil) plants would suggest a smaller and more focused terrorist-type chemical attack with abrin. One possible scenario is that once isolated, abrin could be aerosolized as a dry powder. Studies have found that the overall pattern and time course of damage following inhalation were similar for ricin and abrin and characterized by rapidly progressive and overwhelming pulmonary edema accompanied by acute destructive alveolitis and necrosis/apoptosis of the lower respiratory tract epithelium accounting for the majority of deaths [18,19,20,21]. The clinical manifestations following pulmonary (intranasal) exposure to these toxins were entirely restricted to the lungs and characterized by severe pulmonary edematous inflammation, neutrophil recruitment and development of a proinflammatory cytokine storm [22,23]. Pulmonary (intranasal) ricin and abrin intoxication in mice are similar with regard to pathological features and kinetics. However, despite their resemblance, the ability to protect mice against ricin and abrin intoxication by postexposure antibody-mediated treatment differs drastically. Rabbit-derived polyclonal anti-ricin antibody-based treatment showed almost complete protection a few hours after exposure to a lethal ricin dose; however, when this treatment was delayed to 24 h after intoxication, only one-third of the mice survived [22]. In contrast, the intranasal administration of polyclonal anti-abrin antibodies to mice even as late as 72 h postexposure to a lethal dose of abrin conferred exceedingly high-level protection [23]. Interestingly, the efficient protection by polyclonal anti-abrin antibodies cannot be attributed to the specific neutralization of a particular A or B subunit of the toxin, as antibodies raised against chimeric toxins of either an A_abrin_B_ricin_ or A_ricin_B_abrin_ structure conferred exceptionally high protection levels to mice following intranasal exposure to a lethal dose of abrin [24].

In view of these findings, we characterized and quantified the cellular and molecular changes in murine lung tissue following pulmonary exposure to abrin, as compared to ricin intoxication, to delineate toxin-specific patho-physiologic factors that may play a role in determining the differential ability to protect against abrin and ricin by postexposure antibody administration.

## 2. Results

### 2.1. Differential Binding of Abrin to Lung Cell Populations

**Figure 1:**
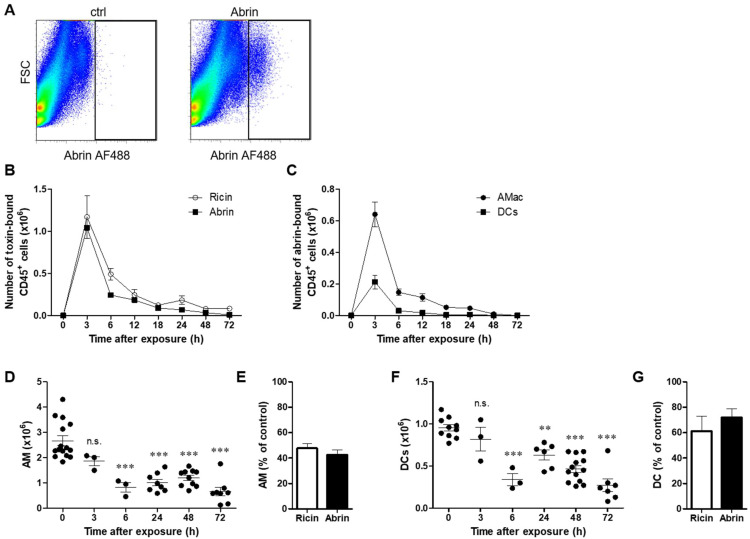
Kinetics of abrin binding to hematopoietic cells and alteration in cell populations in the lung. Mice were intranasally exposed to fluorescent abrin or ricin AF488 (20 or 14 µg/kg body weight, respectively), and lung cells were isolated at 3, 6, 12, 18, 24, 48 and 72 h after exposure and analyzed by flow cytometry for toxin binding by detection of AF488^+^ cells and different cell population counts in the lungs. (**A**) Dot plots represent abrin AF488 staining in lung cells isolated 3 h after abrin intoxication or in cells isolated from control mice. (**B**) Quantification of toxin-bound CD45^+^ cells at different time points following intoxication. A comparison between ricin and abrin intoxications (*n* = 3–9 mice in each group). (**C**) Quantification of abrin-bound AMs and DCs at different time points following abrin intoxication (*n* = 3–6 mice in each group). Quantification of AM (**D**) and DC (**F**) population sizes at different time points following abrin intoxication (10 µg/kg, *n* = 3–15 mice in each group; each point indicates individual mice). The results are depicted as the means ± SEMs. ** *p* < 0.01, *** *p* < 0.001 in comparison to nonintoxicated mice; n.s., not significant. Comparison between abrin and ricin [25] AMs (**E**) and DCs (**G**) (% of control) at 24 h postexposure to toxins.

Previously, we showed that following intranasal intoxication of mice with a lethal dose of ricin, the toxin binds to alveolar macrophages (AMs) and dendritic cells (DCs) of the hematopoietic compartment, as well as to the lung parenchyma, epithelial and endothelial cells [25]. We examined, in a similar system, the interactions between abrin and lung cells after intranasal intoxication of mice with fluorescently labeled abrin (abrin Alexa Fluor 488 (abrin AF488)) at a lethal dose of 2LD_50_. Abrin-associated cells could be visualized by flow cytometry 3 h following exposure to the toxin (Figure 1A). To determine the kinetics of toxin binding to individual cell populations in the lung, mice were intoxicated with abrin or ricin, and lung cells isolated at different time points thereafter were analyzed for toxin binding. In the hematopoietic compartment (CD45^+^ cells), peak binding of both toxins was detected as early as 3 h after intoxication, and at later time points, fewer cells were detected in association with the toxins. The kinetics of CD45^+^ cell binding and the proportion of toxin-bound cells at all examined time points was similar for both toxins (Figure 1B). Among cells of hematopoietic origin, abrin exhibited prompt binding to AMs and DCs, with peak binding at 3 h after intoxication (Figure 1C). To analyze the correlation between the binding of abrin and its ability to eliminate cells by inhibiting protein synthesis, we quantified the number of AMs and DCs at different time points following intoxication. In contrast to ricin pulmonary intoxication, where AMs were significantly reduced 3 h after exposure [25], AMs were heavily reduced 6 h after abrin intoxication, and their numbers stayed low at later time points (24–72 h postexposure, Figure 1D). At 24 h after either ricin or abrin intoxication, the population of AMs comprised only ~40–50% of the initial population of AMs observed in naïve mice (Figure 1E). In a similar manner, the DC population was reduced starting from 6 h post abrin exposure (Figure 1F), in contrast to the significant reduction in these cells already at 3 h after ricin intoxication [25]. One day after intoxication with either of the toxins, the DC population in the lung consisted of only ~60–70% of the initial population measured in non-intoxicated mice (Figure 1G).

**Figure 2:**
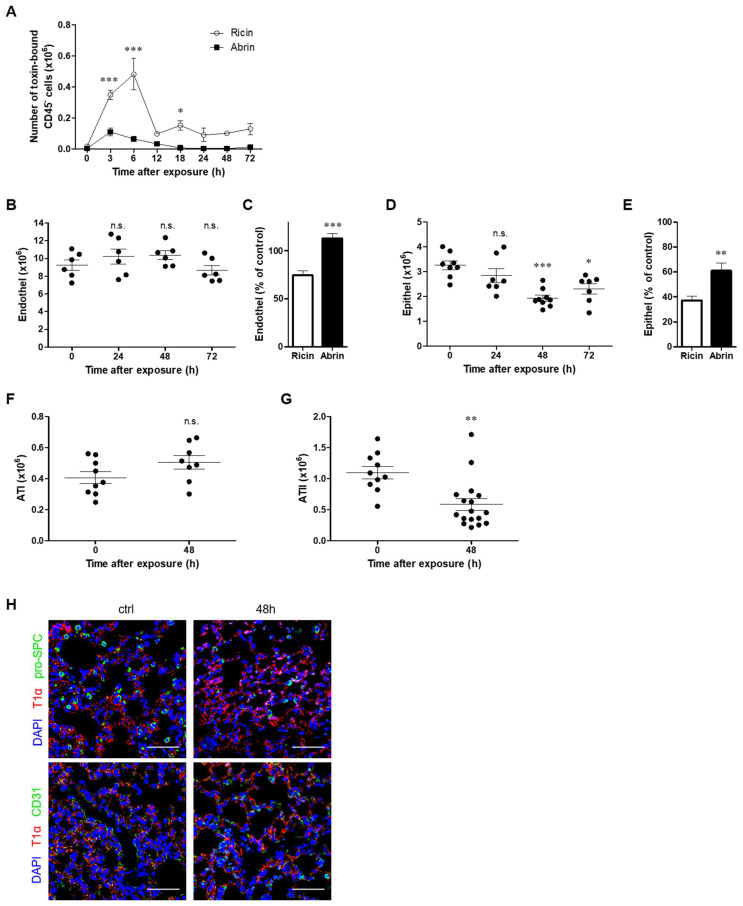
Kinetics of abrin binding to parenchymal cells and alteration in cell populations in the lung. Mice were intranasally exposed to fluorescent abrin or ricin AF488 (20 or 14 µg/kg body weight, respectively), and lung cells were isolated at 24, 48 and 72 h and analyzed by flow cytometry for toxin binding by detection of AF488^+^ cells and for different cell population counts in the lungs. (**A**) Quantification of toxin-bound CD45^−^ cells at different time points following intoxication. Comparison between ricin and abrin intoxications (*n* = 3 mice per group). (**B**) Mice were intranasally exposed to abrin (10 µg/kg), lungs were removed at indicated time points, and endothelial and epithelial (**D**) numbers were determined by flow cytometry (*n* = 6–9 mice in each group). Comparison between the percent of endothelial (**C**) or epithelial (**E**) cells at 48 h post abrin exposure to the percent of these cells at the same time point after ricin intoxication [25]. (**F**) Quantification of alveolar epithelial type I (ATI) and alveolar epithelial type II (ATII) (**G**) cell populations at 48 h post abrin exposure (*n* = 8–17 mice in each group). (**H**) Immunofluorescence analysis of ATI (T1α, red) and ATII (pro-SPC, green) or endothelial cell (CD31, green) staining of lung tissue in nonintoxicated mice (control) versus abrin 48 h postexposed mice (blue, 4′,6-diamidino-2-phenylidole (DAPI) staining of nuclei). Scale bar: 50 µm. The results are depicted as the means ± SEMs. * *p* < 0.05, ** *p* < 0.01, *** *p* < 0.001; n.s., not significant. (**A**,**C**,**E**) Comparison between intoxications at each time point; (**B**,**D**,**F**,**G**) comparison with nonintoxicated mice.

Next, we analyzed the binding of both toxins to the parenchymal cell populations of the lung (CD45^−^ cells). Interestingly, we found that binding to CD45^−^ cells was considerably more pronounced in the case of ricin exposure. Thus, at 3 h after intoxication, twice as many CD45^−^ cells were bound to ricin than to abrin, while at 6 h postexposure, almost 6-fold more cells were associated with ricin than with abrin. Higher levels of toxin binding to lung parenchymal cells following ricin exposure were further observed at all later time points (12–72 h postexposure, Figure 2A). Within the parenchymal compartment, we distinguished between vascular endothelial cells and alveolar epithelial cells. Surprisingly, examination of the endothelial cell population following abrin intoxication did not result in observable damage to the cells, as evidenced by the preserved cell numbers at all tested time points postexposure (Figure 2B). This was in contrast to ricin intoxication, where a ~25% reduction in endothelial cells was detected 48 h postexposure (Figure 2C). Profiling of epithelial cells demonstrated a significant reduction in these cells from 48 h to 72 h after abrin intoxication (Figure 2D). A comparison of ricin and abrin at the same time point showed that the damage to epithelial cells after ricin intoxication was more pronounced, displaying a loss of ~60% of the population, while a reduction in these cells following abrin pulmonary intoxication was no more than ~40% (Figure 2E). Examination of subsets of the epithelial cells demonstrated that although no change was found in alveolar epithelial type I cells after pulmonary abrin intoxication (Figure 2F), the number of alveolar epithelial type II cells decreased significantly by 48 h postexposure (Figure 2G). The reduction in the alveolar epithelial type II population was also manifested with immunohistochemistry by specific labeling of pro-surfactant C (pro-SPC). Immunostaining of the lungs 48 h after abrin intoxication for alveolar epithelial type I cells (anti-T1a) and endothelial cells (anti-CD31) further confirmed no injury to these populations, as comparable staining levels were detected both in nonintoxicated (control) and intoxicated lungs (Figure 2H).

### 2.2. Pulmonary Exposure to Abrin and Ricin Induces Comparable Neutrophil Influx to the Lungs Accompanied by Lung Hyperpermeability

**Figure 3:**
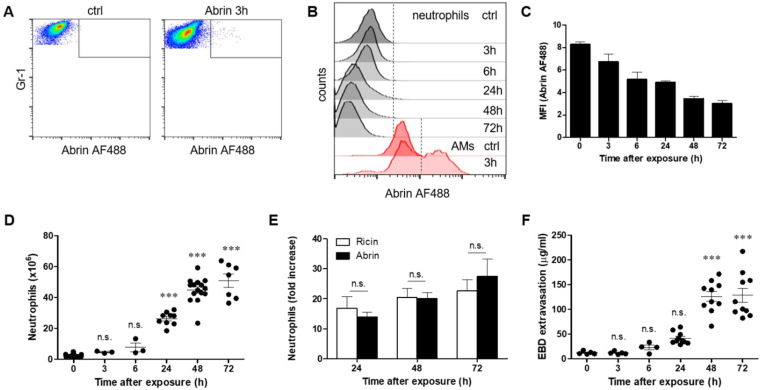
Effect of exposure to abrin on neutrophils and lung permeability. Mice were intranasally exposed to fluorescent abrin AF488 (20 µg/kg body weight), and lung cells were isolated at 3, 6, 24, 48 and 72 h and analyzed for neutrophils by flow cytometry. (**A**) Dot plots represent abrin AF488 staining in neutrophils isolated 3 h after abrin intoxication or in cells isolated from control mice. (**B**) Abrin AF488 binding to neutrophils (black histograms) and AMs (red histograms) at different time points following abrin intoxication. (AMs are autofluorescent in the lungs and exhibit high background in control mice.) (**C**) MFI of abrin AF488 binding to neutrophils (*n* = 3–7 mice in each group). (**D**) Neutrophil count in the lungs at different time points following abrin intoxication. (**E**) Comparison between the increase in neutrophils after abrin and ricin [25] intoxication (*n* = 3–15 mice in each group). (**F**) Lung EBD extravasation following abrin intoxication. Control or abrin-intoxicated mice were intravenously injected with 50 mg/kg EBD at the indicated time points, and lungs were monitored for EBD content (*n* = 4–10 mice in each group). The results are depicted as the means ± SEMs. *** *p* < 0.001; n.s., not significant. In (**D**,**F**), comparison with nonintoxicated mice.

One prominent hallmark of ricin-mediated pulmonary intoxication is the rapid and massive influx of neutrophils to the lungs, where they contribute to the developing inflammation yet may also cause tissue damage, thereby promoting ricin-mediated morbidity [26,27,28]. These neutrophils are refractive to ricin binding [25]. Examination of the binding of abrin to neutrophils in the lungs following intranasal exposure of mice demonstrated that this cell type did not bind abrin at any time point (3–72 h) tested after intoxication (Figure 3A,B). There were no abrin AF488-positive neutrophils in the lungs, which could be appreciated by the low mean fluorescent intensity (MFI) of fluorescent abrin in these cells at all time points after intoxication (Figure 3C). These results are in sharp contrast to AMs, which readily bound abrin, as shown by the increased abrin AF488 intensity (Figure 3B). Next, we monitored neutrophil numbers at different time points after abrin intoxication. Neutrophil counts that comprised ~2 × 106 cells in healthy mice were raised to 26 ± 4 × 106 cells and 50 ± 11 × 106 cells at 24 h and 72 h, respectively, after exposure to abrin (Figure 3D). Alignment between the elevation of neutrophils after ricin and abrin intoxications showed equal recruitment of these cells to the lungs 24–72 h postexposure (Figure 3E). Since uncontrolled massive recruitment of neutrophils may cause tissue damage and promote permeability and edema, we measured lung permeability by the Evans blue dye (EBD) extravasation assay. To this end, mice were intravenously injected with EBD at different time points after intranasal exposure to abrin, lungs were harvested, and EBD was extracted and quantified. Pulmonary EBD levels were found to be elevated significantly at 48 and 72 h post abrin exposure (Figure 3F). These results indicate that similar to ricin pulmonary intoxication, exposure to abrin promotes comparable neutrophil influx into the lungs, which is accompanied by lung hyperpermeability.

### 2.3. Pulmonary Exposure to Abrin Leads to Inferior Impairment of Junction Proteins in the Lungs in Comparison to Ricin

**Figure 4:**
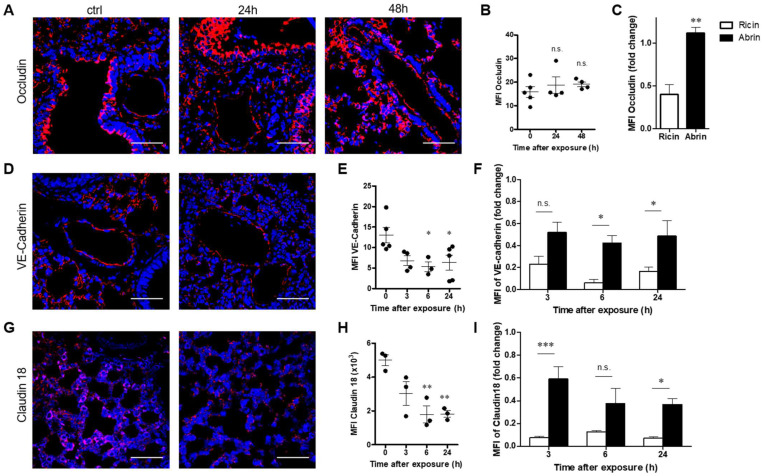
Alterations in occludin, VE-cadherin and claudin 18 in the lungs of abrin-intoxicated mice. Lungs of abrin-intoxicated (10 µg/kg body weight) mice were harvested at the indicated time points, and junction proteins were quantified by immunohistochemical analysis of lung sections. (**A**,**D**,**G**) Confocal microscopy scans of lung sections stained for occludin, VE-cadherin and claudin 18 (red), respectively, and identification of nuclei by DAPI (blue). (**B**,**E**,**H**) Scatterplots represent the immunofluorescence staining intensities of occludin, VE-cadherin and claudin 18, expressed as MFI (*n* = 3–5 mice in each group). Scale bar: 50 µm. (**C**,**F**,**I**) Comparison between the abrin- and ricin-induced reduction in occludin (at 48 h), VE-cadherin and claudin 18 [27]. The results are depicted as the means ± SEMs. * *p* < 0.05, ** *p* < 0.01, *** *p* < 0.001; n.s., not significant. In (**B**,**E**,**H**), comparison with nonintoxicated mice.

The integrity of the alveolar wall barrier depends on the intercellular junctions of the alveolar epithelial and capillary endothelial cells. Junction protein complexes formed by tight junctions (TJs), adherens junctions (AJs) and gap junctions (GJs) stabilize the connections between contiguous cells. Disruption of the integrity of these complexes results in increased permeability and the formation of lung edema [29,30,31]. Indeed, we have previously shown that in the case of ricin pulmonary intoxication, disruption of differential intercellular junction proteins leads to impairment of the alveolar–capillary barrier and to the development of lung edema, which in turn results in the impairment of oxygenation [27,32]. Occludin, a TJ protein, is expressed in the alveoli, bronchial epithelial cells and endothelial cells of blood vessels in healthy lungs. We did not detect any change in occludin expression at 24 and 48 h after abrin intoxication (Figure 4A,B), which was in contrast to ricin intoxication which triggered a two-fold reduction in occludin in the lungs (Figure 4C). Next, we examined the expression of VE-cadherin, which is essential for endothelial barrier integrity. The VE-cadherin level was significantly decreased following abrin exposure (Figure 4D,E), although in comparison to ricin intoxication, which brought nearly complete elimination of VE-cadherin at 3–6 h postexposure, the damage to this protein by abrin was only moderate, even at 24 h post abrin exposure (Figure 4F). Similar to VE-cadherin, claudin 18, which is expressed by alveolar epithelial cells, was also diminished after abrin intoxication (Figure 4G,H), but once again, its reduction was less pronounced than that recorded after pulmonary exposure to ricin (Figure 4I).

**Figure 5:**
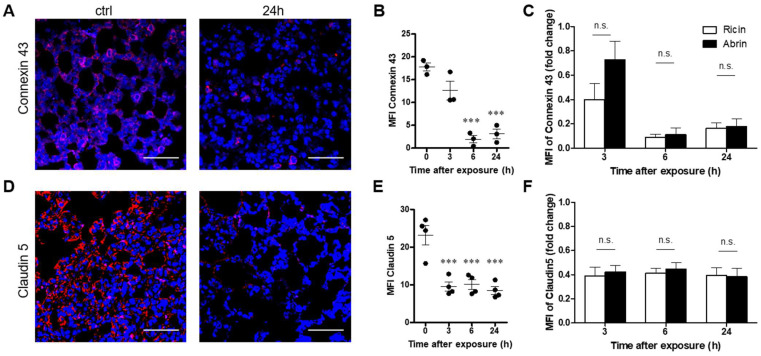
Alterations in connexin 43 and claudin 5 in the lungs of abrin-intoxicated mice. Lungs of abrin-intoxicated (10 µg/kg body weight) mice were harvested at the indicated time points, and junction proteins were quantified by immunohistochemical analysis of lung sections. (**A**,**D**) Confocal microscopy scans of lung sections stained for connexin 43 and claudin 5 (red) and identification of nuclei by DAPI (blue). (**B**,**E**) Scatterplots represent the immunofluorescence staining intensities of connexin 43 and claudin 5, expressed as MFI (*n* = 3–4 mice in each group). Scale bar: 50 µm. (**C**,**F**) Comparison between the abrin- and ricin-induced reduction in connexin 43 and claudin 5 [27]. The results are depicted as the means ± SEMs. *** *p* < 0.001. In (**B**,**E**), comparison with nonintoxicated mice; n.s., not significant.

Examination of connexin 43, a GJ protein, showed that its level was heavily decreased at 6 and 24 h post abrin intoxication (Figure 5A,B). This pronounced decrease in connexin 43 correlated with its elimination after ricin intoxication (Figure 5C). Finally, we examined the expression of claudin 5, which is expressed in both endothelial and airway epithelial cells. The level of claudin 5 was significantly diminished 3 to 24 h after abrin intoxication (Figure 5D,E). As in the connexin 43 case, the reduction in claudin 5 following abrin intoxication was comparable to the level of the protein at the corresponding time points after ricin intoxication, as demonstrated by MFI (Figure 5F).

### 2.4. Pulmonary Exposure to Abrin- and Ricin-Induced Comparable Damage to the Endothelial Glycocalyx

**Figure 6:**
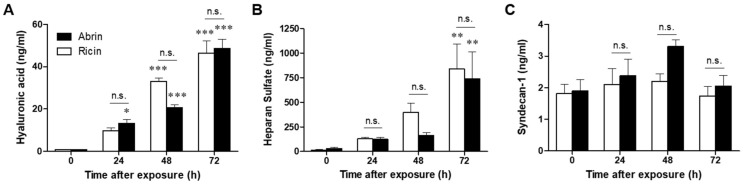
Degradation of the glycocalyx in abrin- and ricin-exposed mice. Levels of soluble hyaluronic acid (**A**), heparan sulfate (**B**) and syndecan-1 (**C**) were determined in the BALF collected from abrin- and ricin (10 or 7 µg/kg body weight, respectively)-exposed mice at the indicated time points (*n* = 4–9 mice in each group). The results are depicted as the means ± SEMs. * *p* < 0.05, ** *p* < 0.01, *** *p* < 0.001; n.s., not significant. The comparison of each column with nonintoxicated mice and between abrin and ricin at each time point.

The endothelial glycocalyx is a complex layer of glycoproteins, proteoglycans and glycosaminoglycans that coat the luminal surface of the vascular endothelium. Hydrated glycosaminoglycans form a thick and rigid endothelial surface layer (ESL) that plays a key role in limiting vascular permeability and regulating leukocyte adhesion [33,34]. Because shedding of the ESL results in hyperpermeability and inappropriate leukocyte adhesion [35], we decided to evaluate the integrity of the ESL after pulmonary exposure to abrin in comparison to ricin intoxication. Mice were intranasally exposed to abrin or ricin, and soluble shed compounds of glycocalyx were analyzed in bronchoalveolar fluid (BALF) harvested at different time points. The detection of soluble glycocalyx compounds, which are indicative of endothelial glycocalyx degradation, negatively correlates with ESL thickness and positively correlates with vascular permeability [30,36]. Syndecan-1, heparan sulfate and hyaluronic acid are the main components whose shedding has been claimed to represent the endothelial glycocalyx state of health. An analysis of the hyaluronic acid levels, a ubiquitous glycosaminoglycan of the ECL, revealed elevated levels of this compound in the BAL of ricin- and abrin-intoxicated mice at 24 h postexposure, which continued to increase at later time points, 48–72 h postexposure. No difference was found in the intensity of the shedding of hyaluronic acid between abrin- and ricin-intoxicated mice (Figure 6A). Monitoring of heparan sulfate, the predominant glycosaminoglycan, demonstrated a marked release of this component at 72 h post abrin and ricin exposures. As in the case of hyaluronic acid, heparan sulfate shedding was similar in response to both toxins at all indicated time points (Figure 6B). Despite the degradation of hyaluronic acid and heparan sulfate, we did not detect shedding of the transmembrane core protein of the glycocalyx, syndecan-1, following either abrin or ricin pulmonary exposure (Figure 6C).

## 3. Discussion

The toxicity of abrin and ricin depends on the route of exposure, with inhalatory exposures considered the most fatal [16]. The clinical manifestation following intranasal exposure of mice to these toxins is the onset of localized yet severe pulmonary edematous inflammation, which is accompanied by massive recruitment of neutrophils to the lungs and onset of a turbulent proinflammatory cytokine storm within this organ [22,37]. Despite the similarity in morbidity and mortality, following pulmonary abrin and ricin intoxications in mice, the ability to protect mice against ricin and abrin intoxications by postexposure antibody-mediated treatment differs radically. In the case of lethal ricin intoxication, rabbit-derived polyclonal anti-ricin antibody-based treatment of the mice at 24 h postexposure resulted in 34% survival rates [22]. When antibody treatment was administered at 48 h postexposure to ricin, protection was no more than marginal [38]. In sharp contrast, the administration of polyclonal anti-abrin antibodies to mice intranasally exposed to a lethal dose of abrin led to very high survival rates (~70–80%), even when the antibodies were applied as late as 72 h after intoxication [22]. This efficient protection by polyclonal anti-abrin antibodies could not be attributed to the neutralization of a single subunit because specific antibodies against the A or B subunits of abrin were equally effective in protecting mice against pulmonary intoxication with chimeric reciprocal toxins harboring one of the subunits of abrin and the other of ricin [24]. These observations indicated that the difference in the protection conferred by anti-abrin and anti-ricin antibodies against abrin and ricin intoxications, respectively, is not related to the difference in the quality of the two antibody preparations. Therefore, in this study, we dissected the differences in abrin and ricin lung pathology following pulmonary exposure of mice to either of the toxins. We found that both toxins bound similarly to hematopoietic cells, especially to AMs and DCs, and triggered their early and persistent elimination from the lungs. These results are in agreement with an earlier study which showed that macrophages are the most sensitive cells to RIPs [39]. In opposition to cells of hematopoietic origin, the toxins differed in their efficiency of binding to parenchymal cells of the lungs. The binding of abrin to CD45^-^ cells was considerably less effective at all tested time points following intoxication. Consequently, following abrin intoxication, there was no direct damage found in the endothelial cell compartment, and the epithelial cell damage that, as in the case of ricin, was mostly confined to the alveolar epithelial type II cells was significantly lower than that observed following ricin intoxication. Supporting these results, we have previously shown that following intranasal intoxication, the in vivo catalytic performance of abrin, i.e., ribosomal depurination of pulmonary tissue, is significantly lower than that observed following ricin intoxication. In particular, the depurination levels of endothelial cells and pulmonary epithelial cells were markedly lower following abrin intoxication in comparison to ricin intoxication [40]. Furthermore, the lesser damage to lung parenchymal cells is in line with an older study that described the histopathology of the lungs in rats and showed that the appearance of apoptosis in the alveolar epithelium was far more marked following inhalation of ricin than abrin [19]. Interestingly, examination of the effect of antibody treatment against the two toxins on lung cell composition following exposure of mice to abrin and ricin showed significant reversion in the cells of hematopoietic origin, neutrophils and macrophages, after both intoxications [23]. However, neither the antibody-based treatment against abrin nor that against ricin conferred any beneficial influence on epithelial cells (data not shown). Since we did not find any repair of the epithelial population in the near term after antibody treatment, we estimate that the intensity of the epithelial damage has a direct effect on the survival of intoxicated and treated mice. As the epithelial damage in the lung is much more extensive after exposure to ricin compared to abrin, the protection ability of anti-ricin antibody treatment is limited.

One prominent hallmark of ricin-mediated pulmonary intoxication is the rapid and massive influx of neutrophils to the lungs [26,27,28]. This uncontrolled recruitment of neutrophils and the overwhelming activation in sterile inflammation, such as in the case of abrin or ricin intoxication, contributes to tissue damage by the release of proteinases, cationic polypeptides, cytokines and reactive oxygen species [41,42]. It has been previously shown that ricin does not bind neutrophils [25,40]. Similarly, we show in this study that infiltrating neutrophils, unlike other cells of hematopoietic origin, did not bind abrin following intoxication.

The dramatic influx over time of toxin-nonbinding neutrophils occurs mainly in the small capillaries spanning the alveolar network [43] and induces indirect lung damage by compromising the permeability of the alveolar–capillary barrier [44]. The kinetics and the extent of neutrophil influx to the lungs were found to be similar following abrin and ricin pulmonary intoxications. However, while the alveolar–capillary barrier integrity was compromised at early stages following ricin intoxication [27], lung permeability following abrin exposure was significantly increased only at later time points (48–72 h). This finding may stem from the relatively reduced levels of irreversible cellular damage inflicted by abrin. Alveolar–capillary barrier permeability is tightly regulated by the molecular interplay of intercellular junction molecules that span the gap between neighboring epithelial and endothelial cells. These junction molecules cooperate in maintaining tissue integrity to limit epithelial and endothelial permeability and to allow for just minimal leakage of fluids into the interstitial compartment [30,45]. We show in this study that in addition to reduced cellular damage, the insult to junction proteins, such as VE-cadherin and claudin 18, following abrin intoxication was less prominent, both at early and late stages, than the damage observed following ricin exposure. Moreover, the tight junction protein occludin, which is reduced at a later stage (48 h) following ricin intoxication [27], was not impaired at all post-abrin-exposure time points. These data may also account for the delayed hyperpermeability of the lungs post abrin exposure in comparison to ricin. However, the pronounced lung hyperpermeability at later time points after exposure to abrin may stem from robust recruitment of neutrophils at these time points and marked impairment in connexin 43 and claudin 5. In addition, although in contrast to ricin, no direct damage to endothelial cells was observed following abrin intoxication, collateral damage to these cells was discerned. In fact, we show in this study that the intensity of the damage to the pulmonary vascular endothelial glycocalyx was comparable following intoxication with the two toxins.

In summary, we propose that the relatively superior performance of the anti-abrin antibody-based treatment and the ability to protect against abrin lethality, but not against ricin, when administered lately following intranasal intoxication in mice is due to the differences in the timing and intensity of the damage to the lung stroma inflicted by abrin and ricin.

## 4. Materials and Methods

### 4.1. Animals

Experiments were performed in accordance with Israeli law and approved by the Institutional Animal Care and Use Committee (IACUC) of the Israel Institute for Biological Research (Ness-Ziona, Iarael). Treatment of animals was in accordance with regulations outlined in the USDA Animal Welfare Act and the conditions specified in the National Institute of Health Guide for Care and Use of Laboratory Animals. Female CD-1 mice (27–32 g) were purchased from Charles River Laboratories Ltd., Margate, UK. Mice were housed in filter-top cages in an environmentally controlled room and maintained at 21 ± 2 °C and 55 ± 10% humidity. Lighting was set to mimic a 12/12 h dawn-to-dusk cycle. Mice were housed in a purpose-built animal holding facility for 4–8 days prior to the beginning of the experiment. Animals were allowed access to water ad libitum and 4% body weight food per day.

### 4.2. Fluorescent Toxin Labeling and Intoxication

Abrin and ricin were purified as previously described [22,23] and conjugated (1 mg) with the Alexa Fluor^TM^ 488 protein labeling kit (Molecular probes, Thermo Fisher Scientific, Waltham, MA, USA) according to the manufacturer’s instructions. The cytotoxicity of labeled toxin (~5 dye/protein (mol/mol)) was determined in a cell-based assay developed in the past [46]. Briefly, labeled abrin or ricin was added to HEK-293 cell cultures, which secrete the enzyme acetylcholinesterase (AChE) in a constitutive manner. Secreted AChE was measured in the cell growth medium at 18 h postexposure, and activity levels were compared to those measured for nonlabeled toxin. We determined a ~2-fold reduction in the toxicity of the labeled toxins. Intranasal intoxication with toxin at a 2LD_50_ dose (unlabeled and labeled ricin or abrin, 7 and 14 µg/kg or 10 and 20 µg/kg, respectively) was applied (2 × 25 µL).

### 4.3. Flow Cytometry

Lungs were minced into small pieces and subjected to enzymatic digestion with 4 mg/mL collagenase D (Roche, Mannheim, Germany) for 2 h at 37 °C. The tissues were then meshed through a 40 μm cell strainer, and red blood cells were lysed with red blood cell lysis buffer (Sigma–Aldrich, Rehovot, Israel). For staining, cell suspensions were stained with CD45 (clone 30-F11), CD11b (M1/70), Ly6G (1A8), CD11c (N418), MHC class II (M5/114), Siglec F (S17007L), CD31 (390), CD326 (G8.8), T1α (8.1.1) and proSPC (Millipore, Temecula, CA, USA) followed by allophycocyanin (APC) donkey anti-rabbit IgG (Jackson ImmunoResearch, West Grove, PA, USA). All antibodies were purchased from Biolegend (San Diego, CA, USA) unless otherwise indicated. Neutrophils were identified as CD45^high^, Ly6G^high^ and CD11b^high^; AMs as CD45^high^, autofluorescent, Siglec F^high^ and CD11c^high^; DCs as CD45^high^, Siglec F^neg^ and CD11c^high^ and MHC class II^high^; endothelial cells as CD45^neg^ and CD31^high^; epithelial cells as CD45^neg^ and CD326^high^; alveolar epithelial cells type I as CD45^neg^ and CD31^neg^, CD326^high^ and T1α^high^; epithelial cells as CD45^neg^ and CD326^high^; and alveolar epithelial cells type II as CD45^neg^ and CD31^neg^, CD326^high^ and proSPC^high^ cells. Flow cytometry was performed on a FACSCalibur (BD Biosciences, San Jose, CA, USA) and analyzed using FlowJo software v.10.8.0 (Tree Star, Ashland, OR, USA).

### 4.4. Immunohistochemistry

Lungs were collected and fixed in 4% buffered formaldehyde in PBS pH 7.2–7.4 (Bio Lab, Jerusalem, Israel) for 2 weeks. Sections of 5 µm were prepared after paraffin embedding using an RM 2255 microtome (Leica, Nussloch, Germany). Antigen retrieval was performed by incubation in Target Retrieval Solution (S1700, DAKO, Carpinteria, CA, USA, 30 min, 95 °C). After blocking in 5% BSA in PBS, slides were incubated (overnight, 4 °C) with purified anti-CD31 (390, Biolegend, San Diego, CA, USA), proSPC (Millipore, Temecula, CA, USA), podoplanin (T1α, 8.1.1, Biolegend), VE-cadherin (ab33168), claudin 5 (ab15106), connexin 43 (ab117843), occludin (ab31721) or claudin 18 (ab203563) (Abcam, Cambridge, MA, USA). Alexa Fluor 594- or 488-coupled donkey anti-rabbit or Alexa Fluor 594-coupled goat anti-Armenian hamster antibodies were used for detection (Molecular probes®, Thermo Fisher Scientific, Carlsbad, CA, USA). For nuclear staining, slides were mounted with Prolong® Gold antifade reagent containing DAPI (Molecular probes®, Thermo Fisher Scientific, Carlsbad, CA, USA). Analysis was performed using an LSM 710 confocal scanning microscope (Zeiss, Jena, Germany) equipped with the following lasers: argon multiline (458/488/514 nm), diode 405 nm, DPSS 561 nm and helium-neon 633 nm.

### 4.5. Permeability Analysis

Lung permeability was determined by the Evans blue dye (EBD) extravasation method as follows: EBD (7.5 mg/mL, Sigma–Aldrich, Rehovot, Israel) was injected intravenously into mice at a dose of 50 mg/kg and allowed to circulate for 1 h. Mice were then anesthetized, and the lungs were perfused by cutting the left atrium and flushing with 5 mL PBS through the right ventricle. The lungs were removed, and EBD was extracted by incubation of the tissues in 0.5 mL of formamide (Sigma–Aldrich, Rehovot, Israel) at 60 °C for 24 h. The EBD optical density in the supernatant was measured at 620 nm in a spectrophotometer (Molecular Devices, Sunnyvale, CA, USA), and the total amount of dye was calculated by means of a standard calibration curve.

### 4.6. Analysis of Glycocalyx Shedding

BALF was performed by flushing the lungs with 1 mL of PBS using a tracheal cannula. The BALF was centrifuged at 950× *g* at 4 °C for 10 min, and the supernatants were collected and tested for soluble heparan sulfate, hyaluronic acid and syndecan-1 levels using an LSBio (Seattle, WA, USA) Mouse Heparan Sulfate ELISA kit (LS-F39210), R&D Systems (Abingdon, UK) Porcine/Mouse Quantikine Hyaluronan Immunoassay kit (DHYAL0) and Diaclone (Besancon Cedex, France) Murine sCD138 (Syndecan-1) ELISA kit (860.090.096) according to the manufacturer’s instructions.

### 4.7. Statistical Analysis

All statistical analyses were conducted with GraphPad Prism software (version 5.01, GraphPad Software Inc., La Jolla, CA, USA, 2007). Data are presented as the means ± SEMs. Significance was assessed by Student’s t test, and for multiple comparisons, one-way analysis of variance (ANOVA) followed by Tukey’s multiple comparisons test or two-way ANOVA with Bonferroni correction was used for planned comparisons. Differences were considered significant at *p* < 0.05. Points in graphs indicate individual mice.

## Data Availability

Not applicable.
